# Resveratrol prevents p53 aggregation *in vitro* and in breast cancer cells

**DOI:** 10.18632/oncotarget.25631

**Published:** 2018-06-26

**Authors:** Danielly C. Ferraz da Costa, Nathali P.C. Campos, Ronimara A. Santos, Francisca Hildemagna Guedes-da-Silva, Mafalda Maria D.C. Martins-Dinis, Letícia Zanphorlin, Carlos Ramos, Luciana P. Rangel, Jerson L. Silva

**Affiliations:** ^1^ Departamento de Nutrição Básica e Experimental, Instituto de Nutrição, Universidade do Estado do Rio de Janeiro, Rio de Janeiro 20550-013, RJ, Brazil; ^2^ Instituto Nacional de Ciência e Tecnologia de Biologia Estrutural e Bioimagem, Universidade Federal do Rio de Janeiro, Rio de Janeiro 21941-902, RJ, Brazil; ^3^ Programa de Biologia Estrutural, Instituto de Bioquímica Médica Leopoldo de Meis, Universidade Federal do Rio de Janeiro, Rio de Janeiro 21941-902, RJ, Brazil; ^4^ Instituto de Química, Universidade de Campinas, Campinas 13083-970, SP, Brazil; ^5^ Departamento de Análises Clínicas e Toxicológicas, Faculdade de Farmácia, Universidade Federal do Rio de Janeiro, Rio de Janeiro 21941-902, RJ, Brazil

**Keywords:** amyloid aggregation, p53, cancer, aggregation, resveratrol

## Abstract

One potential target for cancer therapeutics is the tumor suppressor p53, which is mutated in more than 50% of malignant tumors. Loss of function (LoF), dominant negative (DN) and gain of function (GoF) mutations in p53 are associated with amyloid aggregation. We tested the potential of resveratrol, a naturally occurring polyphenol, to interact and prevent the aggregation of wild-type and mutant p53 *in vitro* using fluorescence spectroscopy techniques and in human breast cancer cells (MDA-MB-231, HCC-70 and MCF-7) using immunofluorescence co-localization assays. Based on our data, an interaction occurs between resveratrol and the wild-type p53 core domain (p53C). In addition, resveratrol and its derivatives pterostilbene and piceatannol inhibit mutant p53C aggregation *in vitro*. Additionally, resveratrol reduces mutant p53 protein aggregation in MDA-MB-231 and HCC-70 cells but not in the wild-type p53 cell line MCF-7. To verify the effects of resveratrol on tumorigenicity, cell proliferation and cell migration assays were performed using MDA-MB-231 cells. Resveratrol significantly reduced the proliferative and migratory capabilities of these cells. Our study provides evidence that resveratrol directly modulates p53, enhancing our understanding of the mechanisms involved in p53 aggregation and its potential as a therapeutic strategy for cancer treatment.

## INTRODUCTION

In recent decades, cancer has emerged as a major public health problem, and approximately 13 million cancer-related deaths are estimated to occur worldwide by 2030 [1–3]. Generating effective cancer therapeutics is a substantial challenge that requires precision medicine strategies. Although the molecular mechanisms of carcinogenesis are not fully understood, involvement of the tumor suppressor p53 is considered crucial, as p53 plays an essential role preventing cancer development by inducing cell cycle arrest and/or apoptosis in response to genotoxic stress [4–7].

The p53 protein is a tetrameric nuclear phosphoprotein containing 393 amino acids and three functional regions: the N-terminal activation domain, which interacts with a variety of proteins; the C-terminal domain responsible for oligomerization; and the core domain (p53C), which encodes the sequence-specific DNA-binding region of the protein [8]. Mutations in the p53 gene (*TP53*) are frequently associated with an increased susceptibility to develop cancer, and inactivation of p53-regulated pathways has been described in over 50% of all human cancers [7].

Recently, the intracellular aggregation of mutant p53 protein has been shown to inactivate p53, aggravating or inducing malignancy [9–15]. As shown in our previous studies, wild-type and mutant p53 core domains form β-sheet-rich fibrillar aggregates under mild denaturing conditions. In addition, these aggregates exert a cytotoxic effect on cultured macrophages [16]. Amyloid aggregates form in regions of the protein other than p53C [10]. For example, aggregates of the N-terminal transactivation domain form at a low pH [17]. However, according to our previous study, the interaction between p53 and small cognate DNA stabilizes p53C and the full-length p53 protein, thus preventing aggregation [12]. Furthermore, a mutant form of p53 co-localizes with amyloid-like protein aggregates in breast cancer biopsies and in tumor cell lines [13]. Aggregates of mutant p53 have been suggested to induce the co-aggregation of wild-type p53 and other p53 homologues, including p63 and p73 [14]. The aggregation of p53 into a mixture of oligomers and fibrils sequesters the native protein into an inactive conformation, which is a typical prion-like behavior [15]. Thus, p53 aggregation may participate in some cancers through a mechanism similar to amyloid diseases.

Resveratrol (*trans*-3,4’,5-trihydroxystilbene), a natural stilbene present in many plants, including grapes, berries and peanuts, is a promising alternative cancer chemopreventive agent. Resveratrol regulates many cellular targets involved in cancer signaling pathways [18–24]. This bioactive compound activates many proteins that participate in cell cycle arrest and apoptosis, such as the tumor suppressor p53 [25, 26]. Several other stilbene compounds that are naturally present in foods have important biological properties. Pterostilbene (3,5-di-methoxy-40-hydroxystilbene) and piceatannol (3,3’,4’,5-trans-tetrahydroxystilbene), which are structurally similar to resveratrol, are also promising cancer chemopreventive agents [27, 28].

Resveratrol induces p53-dependent cell death in a variety of cell lines [29–36]. Although several indirect mechanisms for p53 activation by resveratrol have been proposed, this bioactive compound has not been shown to directly modulate p53. Resveratrol might exert its biological effects by interacting with specific proteins, denoted *resveratrol target proteins* (RTPs) [37]. Based on data from thermodynamic analyses and molecular modeling studies, resveratrol binds to the plasma proteins albumin and hemoglobin through hydrophobic interactions and hydrogen bonds, respectively [38]. Binding of this compound to β-globulin [39] and plasma lipoproteins has also been reported [40]. Winter and co-workers have shown that resveratrol inhibits islet amyloid polypeptide (IAPP) aggregation, a mechanism involved in the pathogenesis of type-II diabetes mellitus [41]. Additionally, Pineda-Sanabria *et al.* demonstrated the structure of *trans*-resveratrol bound to the calcium-binding protein troponin C, which they solved using nuclear magnetic resonance (NMR) spectroscopy [42].

The specific cellular targets that might be associated with the chemopreventive activity of resveratrol require further study. Thus, we examined whether this bioactive compound directly modulates p53 by investigating a potential interaction between resveratrol and p53C. In addition, we tested the potential of resveratrol and its derivatives to prevent p53 protein aggregation *in vitro* and in breast cancer cells. These findings may help elucidate crucial mechanisms that should be considered when developing novel strategies for preventing p53 protein aggregation as a potential cancer therapy.

## RESULTS

### Resveratrol suppresses the intrinsic fluorescence of wild-type p53C

**Figure 1:**
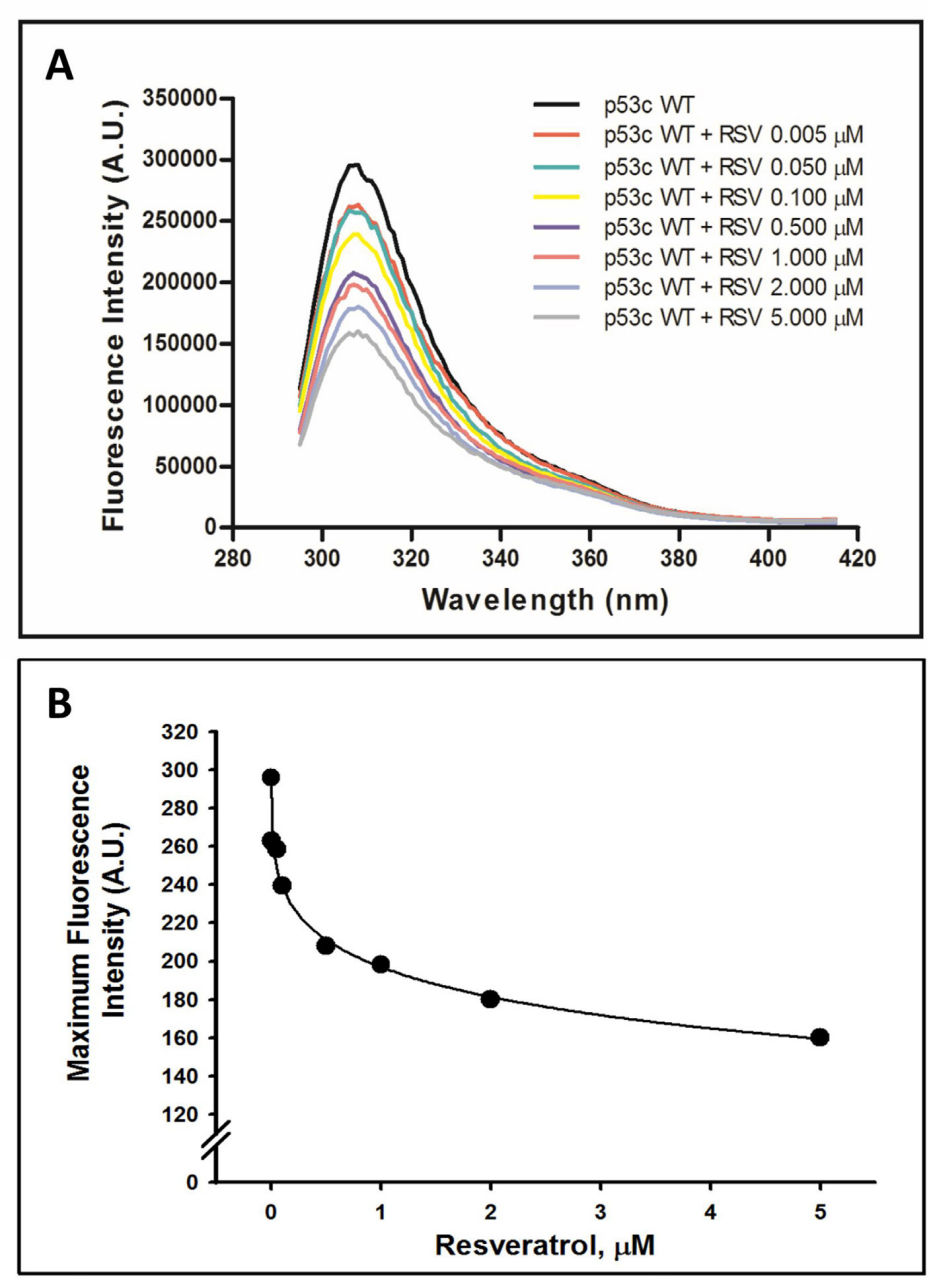
Effect of resveratrol on the tertiary structure of the wild-type p53 core domain (p53C) Intrinsic fluorescence spectra of tyrosine residues were collected at 25° C, and the protein concentration was 10 µM. Samples were excited at 278 nm, and the emission spectra were collected from 295 to 415 nm. The data are representative of six independent experiments (**A**). Maximum fluorescence intensity of the wild-type p53 core domain (p53C) as a function of resveratrol concentrations. Data were obtained from the intrinsic fluorescence emission spectra of p53C in the presence of resveratrol, shown in panel A (*n* = 6) (**B**).

Although p53C is primarily known as a DNA-binding domain, other molecules interact with this region of the protein [43]. Because p53 has been implicated in the anticancer properties of resveratrol, we examined whether this bioactive compound directly modulates p53C by promoting changes in tyrosine fluorescence intensity. Figure 1A shows the fluorescence spectra of wild-type p53C in the presence of different concentrations of resveratrol (0.005–5 µM). p53C displays an emission peak at approximately 308 nm with an excitation wavelength of 278 nm. The maximum emission peaks decreased, indicating that resveratrol caused the concentration-dependent quenching of p53C intrinsic fluorescence (Figure 1B).

### Resveratrol inhibits the aggregation of wild-type and R248Q p53C *in vitro*

**Figure 2:**
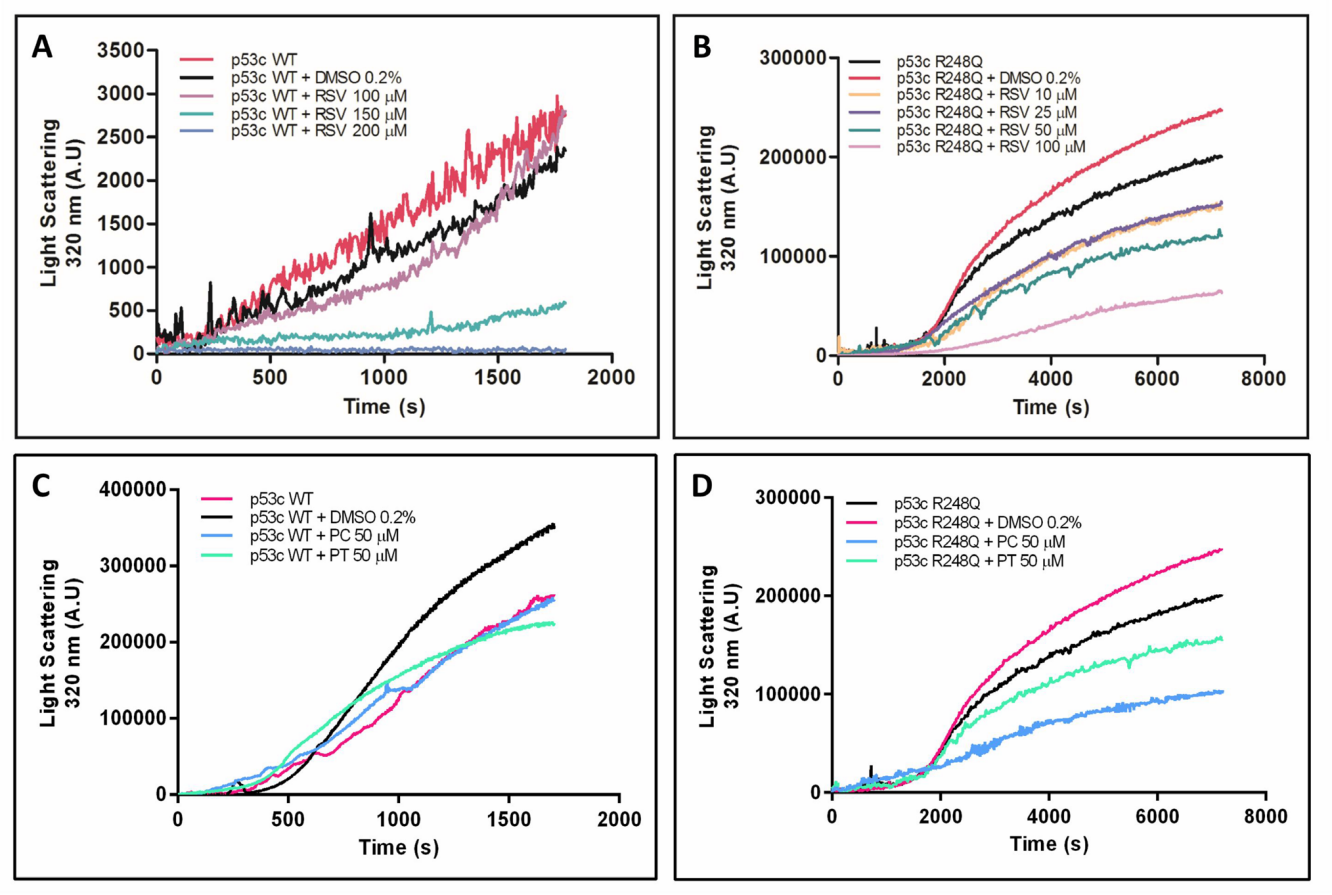
Aggregation kinetics of wild-type p53C and p53C R248Q in the presence of resveratrol (**A** and **B**), pterostilbene and piceatannol (**C** and **D**). Proteins (5 µM) were incubated with different concentrations of resveratrol or its analogues at 37° C, and the aggregation kinetics were monitored by measuring light scattering for (A) 30 or (B–D) 120 min.

Previous studies have reported the ability of wild-type and mutant p53C to form aggregates *in vitro* and in tumor cells [9, 13–15, 44–46]. First, we monitored the aggregation kinetics of the wild-type and R248Q mutant form of p53C at 37° C by measuring light scattering values at 320 nm to evaluate the effect of resveratrol and its derivatives, pterostilbene and piceatannol, on p53C aggregation (Figure 2). Both wild-type and R248Q p53C formed aggregates when incubated for 30 min at 37° C, which was demonstrated by the increase in the light scattering values. Under the same experimental conditions, the R248Q mutant displayed more aggregates than did wild-type p53C (Figure 2A–2D). Resveratrol inhibits the aggregation of both wild-type and R248Q p53C in a concentration-dependent manner (Figure 2A and 2B, respectively). This effect is more pronounced for the R248Q mutant than for wild-type p53C, as lower concentrations of resveratrol were required to inhibit the aggregation of the mutant protein compared to wild-type p53C. Pterostilbene and piceatannol (50 µM) also reduced the aggregation of p53 R248Q but to a lesser extent than resveratrol (Figure 2D), and did not change the aggregation of wild-type p53C (Figure 2C). Resveratrol has been shown to inhibit aggregation of other amyloidogenic proteins, such as the islet amyloid polypeptide and the protein transthyretin [41, 48]. However, this effect is not non-specific. For example, resveratrol does not affect the thermal aggregation of BSA (Supplementary Figure 1).

### Resveratrol inhibits p53 aggregation in human breast cancer cells and in xenograft tumors

**Figure 3:**
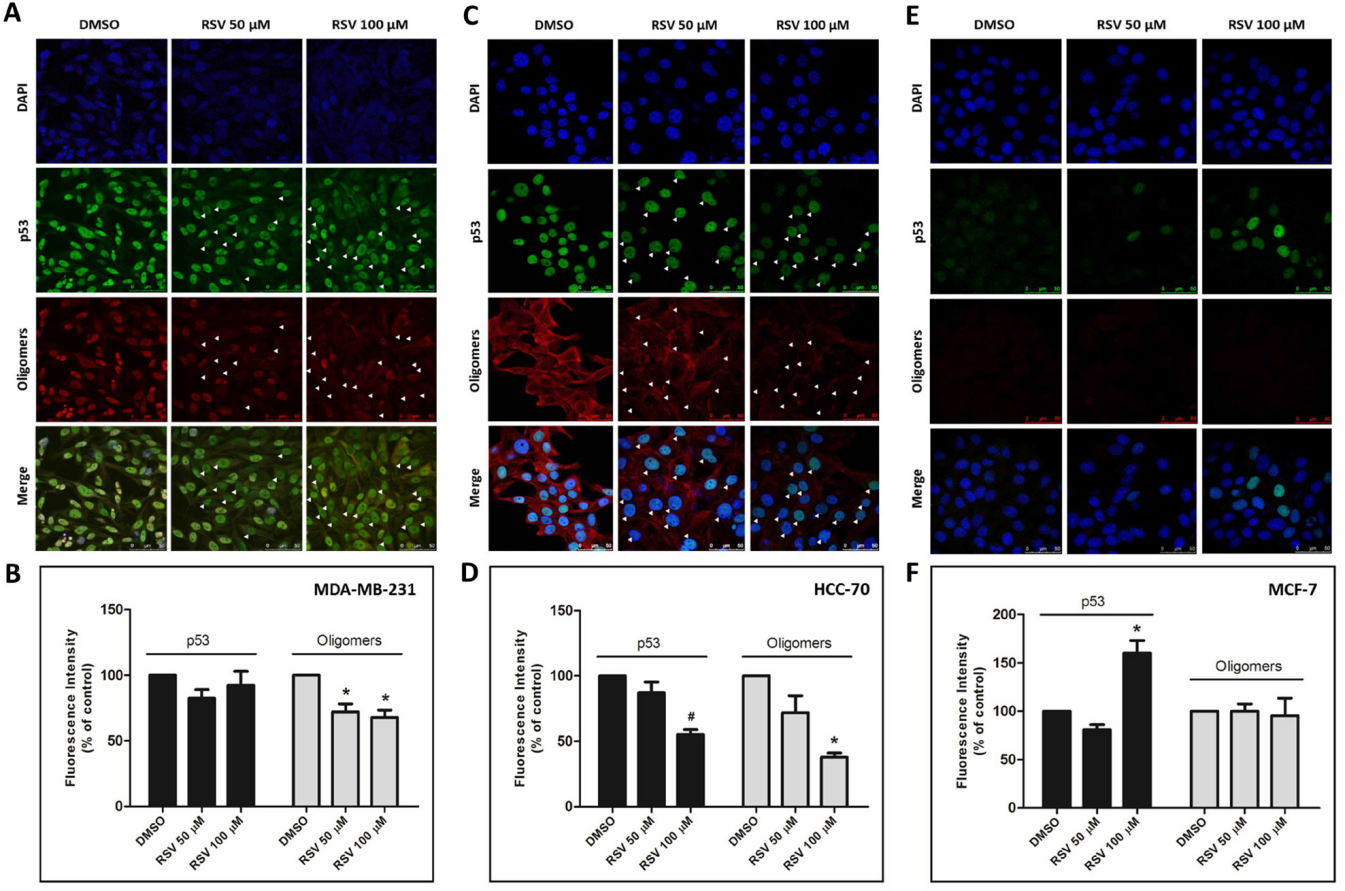
Resveratrol prevents p53 aggregation in breast cancer cells (**A**) MDA-MB-231, (**C**) HCC-70 and (**E**) MCF-7 cells were incubated with 50 and 100 µM resveratrol for 24 h. The cells were simultaneously labeled with a mouse monoclonal anti-human p53 protein DO-1 primary antibody (1:200) and an anti-oligomer A11 (1:1000) primary antibody, as indicated. Next, the cells were incubated with Texas Red 561- and IRDye 680 LT-conjugated secondary antibodies at room temperature in a dark chamber. Finally, the cells were washed and analyzed using confocal laser scanning microscopy. Total fluorescence intensity quantification was performed using ImageJ software, version 1.43r (NIH, USA) (**B**, **D** and **F**).

In our previous study, we showed predominant nuclear co-localization of mutant p53 (R280K) with amyloid aggregates in MDA-MB-231 human breast cancer cells [15]. Here, exposure to resveratrol at 50 and 100 µM for 24 h promoted a significant (*p* < 0.005) reduction in nuclear p53 aggregate formation (Figure 3A, white arrows, and 3B). However, the resveratrol-related compounds pterostilbene and piceatannol did not effectively reduce p53 aggregate formation in MDA-MB-231 cells (Supplementary Figure 2, supplementary Data). We also investigated the anti-aggregation potential of resveratrol on HCC-70, a highly invasive human breast ductal carcinoma that expresses the R248Q form of mutant p53. The results showed that p53 amyloid aggregates in HCC-70 were distributed throughout the cells and were not predominantly concentrated in the nucleus, as in MDA-MB-231. Resveratrol also significantly (*p* < 0.005) reduced the formation of these aggregates at 100 µM, corroborating the previous *in vitro* results (Figure 3C and 3D). In these cells, resveratrol also decreased p53 mutant R248Q levels. In MCF-7 cells (p53 wild-type), which exhibit very low levels of amyloid aggregates compared to p53 mutant cell lines, resveratrol increased p53 protein at 100 µM but had no effect on p53 aggregate formation (Figure 3E and 3F). Treatment with resveratrol (25 mg/kg daily IP for 10 days) in a nude mice xenograft model of breast cancer using MDA-MB-231 cells, in which tumors were allowed to grow for nine weeks, also showed a reduction of mutant p53 and p53 amyloid aggregate levels, as observed in immunohistochemistry experiments (Figure 5). In addition, tissues stained with hematoxylin and eosin revealed blood vessels in the lungs of untreated animals, possibly due the establishment of metastases, which were completely suppressed by resveratrol treatment. No morphological differences were observed in the liver and in tumor tissues between these groups (Supplementary Figure 3).

**Figure 4:**
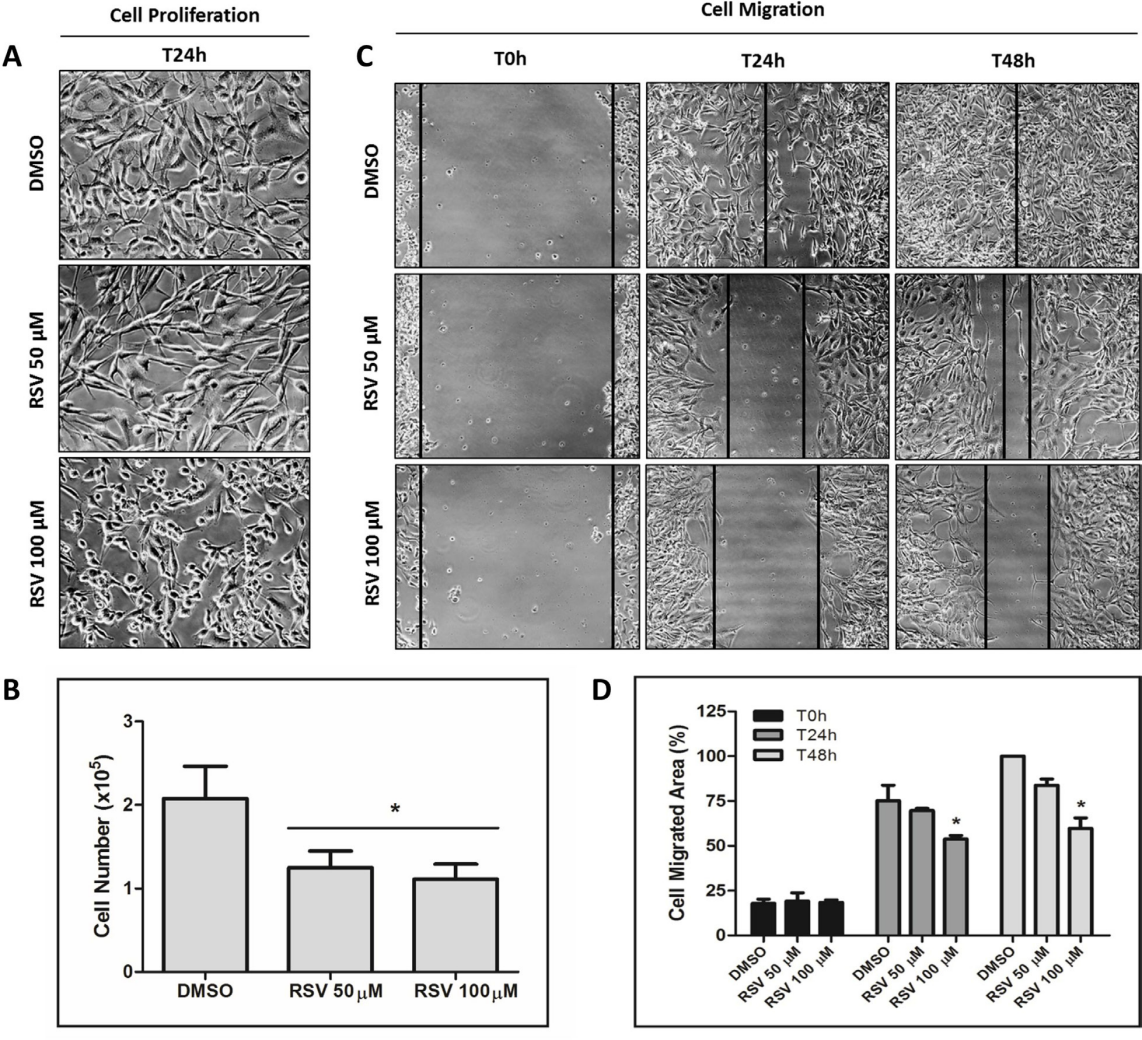
Inhibition of MDA-MB-231 cell proliferation and migration by resveratrol MDA-MB-231 cells were incubated with 50 or 100 µM resveratrol for 24 h and 48 h for cell proliferation (**A** and **B**) and migration (**C** and **D**) assays, respectively.

**Figure 5:**
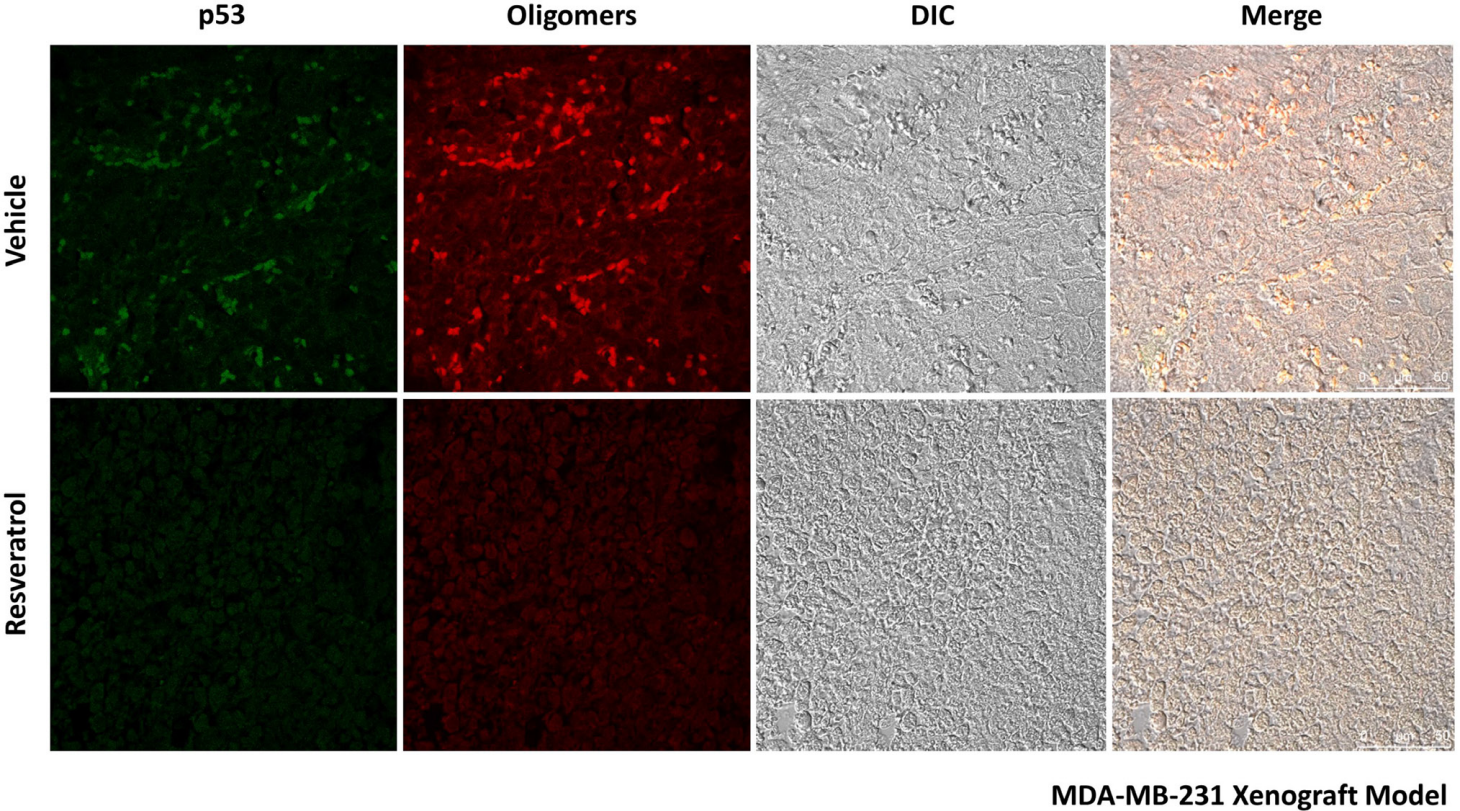
Resveratrol reduces mutant p53 and p53 amyloid aggregates in the tumor tissues of a nude mouse xenograft model of breast cancer using MDA-MB-231 cells Animals were treated with 25 mg/kg daily IP for 10 days. Tissue sections were simultaneously labeled with a mouse monoclonal anti-human p53 protein DO-1 primary antibody (1:200) and an anti-oligomer A11 primary antibody (1:500), as indicated. Next, sections were incubated with anti-mouse Alexa Fluor 488 and anti-rabbit Alexa Fluor 546 secondary antibodies (1:1000).

### Resveratrol reduces MDA-MB-231 human breast cancer cell migration and proliferation

To investigate whether the decreased p53 aggregation caused by resveratrol has an impact on tumorigenicity, we performed cell proliferation and cell migration assays in MDA-MB-231 cells with the same conditions that causes resveratrol inhibition of p53 amyloid aggregates. We observed a significant reduction (*p* < 0.005) in cell proliferation and migration capability in cells exposed to resveratrol (Figure 4A–4D).

## DISCUSSION

In this study, we hypothesized that the tumor suppressor protein p53 is directly modulated by resveratrol. The data demonstrated an *in vitro* interaction between this bioactive compound and wild-type p53C. Additionally, resveratrol inhibited the aggregation of wild-type p53C and the R248Q p53 mutant, a cellular process that might drive malignancy as a result of a p53 LoF mutation.

Several mechanisms for p53 activation by resveratrol have been previously reported. Resveratrol induces a p53-dependent response in a variety of tumor cell lines and in animal carcinogenesis models [29–36]. Although resveratrol binds certain proteins [37–40, 42], this bioactive compound has not been shown to interact with p53 *in vitro*.

We tested the effects of resveratrol on wild-type p53C using fluorescence spectroscopy techniques. Resveratrol promoted a concentration-dependent decrease in tyrosine fluorescence at 278 nm, suggesting potential quenching of p53C intrinsic fluorescence. Based on these data, p53C and resveratrol interact *in vitro*. Resveratrol has been shown to bind to a variety of proteins, including albumin, hemoglobin, β-lactoglobulin plasma lipoproteins, troponin C [37–40, 42] and other molecules, such as DNA [47]. Diethylstilbestrol, a synthetic estrogen with a molecular structure similar to resveratrol, binds to and stabilizes the homotetrameric plasma protein transthyretin, thereby preventing the formation of amyloid aggregates [48].

According to previous reports, p53 aggregation is associated with some cancers through a mechanism similar to amyloid diseases [15, 49–52]. As shown here, resveratrol prevented p53C aggregation *in vitro* in a dose-dependent manner (Figure 2A and 2B), without affecting BSA aggregation, used as a control protein (Supplementary Figure 1). The inhibitory effects of resveratrol on the p53 R248Q mutant were more pronounced than those on wild-type p53C. Because aggregation occurred in the R248Q mutant more rapidly and to a greater extent than that in wild-type p53C, we propose that resveratrol is an alternative bioactive compound that effectively blocks p53 aggregation *in vitro*. A recent report showed that a peptide designed to bind to the amyloidogenic segment of p53 was able to inhibit mutant p53 aggregation [53]. Aggregation of mutant p53 has emerged as a potential target against cancer [54].

Although the core domain of p53 is primarily known to be a DNA-binding domain, other molecules interact with this region of the protein. PRIMA-1, a p53 reactivating drug, has been previously shown to rescue the function of mutant p53 proteins by interacting with p53C, thus enabling tumor cells to undergo apoptosis [43].

Resveratrol dramatically reduced p53 aggregates in tumor cell lines containing mutant p53 (MDA-MB-23 and HCC-70 cells) but not in the wild-type p53 cell line MCF-7. These results were corroborated by pathophysiological effects. Resveratrol inhibits mutant p53 aggregation in MDA-MB-231 cells and also reduced cell migration and proliferation. These functional data confirm that resveratrol has important anti-neoplastic effects (Figure 4). Nude mice with xenografts of MDA-MB-23 cells treated with resveratrol also demonstrated a significant decrease in mutant p53 aggregation. Ongoing studies are currently being performed with a greater number of animals to evaluate the effects on the tumors not only using resveratrol but some chemically modified resveratrol compounds.

**Figure 6:**
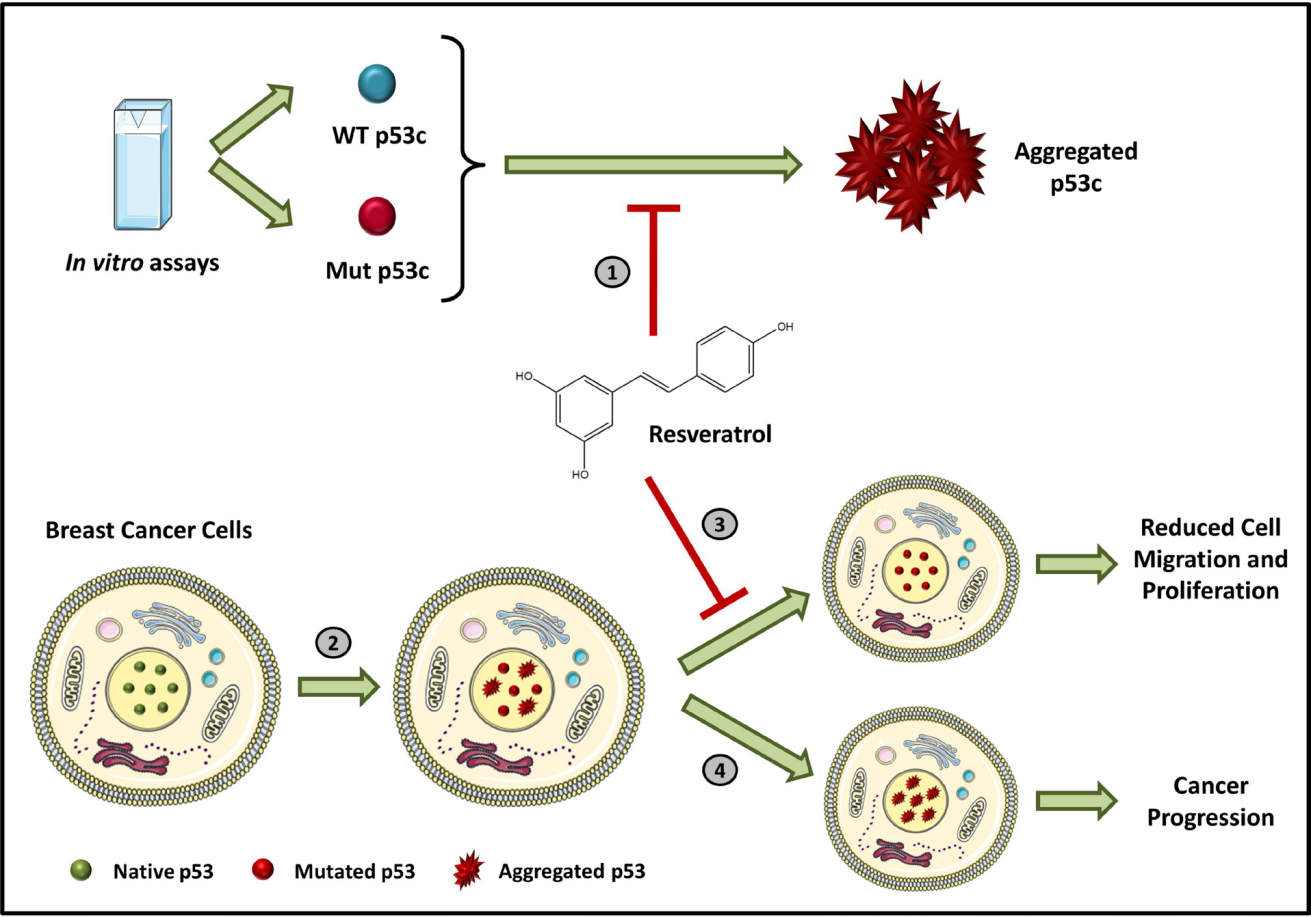
Inhibition of p53 amyloid aggregation by resveratrol (**1**) Resveratrol inhibits wild-type and mutant p53C aggregation *in vitro*. (**2**) Mutations in p53 protein in cancer cells are directly associated with amyloid aggregation. (**3**) Resveratrol impairs the formation of p53 amyloid-aggregates in MDA-MB-231 and HCC-70 human breast cancer cells and reduces cell migration and proliferation. (**4**) However, in the absence of this bioactive compound, massive nuclear accumulation of the aggregates occurs, increasing cancer aggressiveness and progression.

To the best of our knowledge, this study is the first to show that resveratrol inhibits p53 aggregation (Figure 6), supporting the hypothesis that these molecules interact. Resveratrol likely stabilizes p53C and the mutant R248Q, impairing aggregation. The interactions of resveratrol with other functional regions of p53, such as the C-terminal and N-terminal domains, should be considered and warrant further investigation. This study provides evidence that resveratrol directly modulates p53 and enhances our understanding of the mechanisms involved in p53 aggregation as a therapeutic strategy for cancer treatment. Our data indicate that resveratrol is a highly promising lead compound targeted against mutant p53 aggregation.

## MATERIALS AND METHODS

### Reagents

All reagents used in this study were of analytical grade. Trans-resveratrol (3,5,4’-trihydroxy-trans-stilbene), pterostilbene (3,5-di-methoxy-40-hydroxystilbene) and piceatannol (3,3’,4’,5-trans-tetrahydroxystilbene) were purchased from Sigma-Aldrich (St. Louis, MO, USA). Distilled water was filtered and deionized with a Millipore^®^ (Darmstadt, Germany) water purification system.

### Sub-cloning, expression and purification of wild-type p53C and R248Q

Sequences for p53C and R248Q p53C (encoding amino acid residues 94 to 312) were sub-cloned, expressed, and purified using previously described methods [16]. Protein samples were stored in 50 mM Tris–HCl (pH 7.2), 150 mM NaCl, 5 mM dithiothreitol (DTT) and 5% (v/v) glycerol in liquid nitrogen.

### Fluorescence spectroscopy and light scattering analysis

Intrinsic fluorescence based on the tyrosine emission spectrum was analyzed using an ISS/K2 spectrofluorometer (ISS, Champaign, IL, USA). The samples (wild-type p53C and R248Q mutant, 7.5 µM) were excited at 278 nm, and emission spectra were collected from 295 to 415 nm. Light scattering experiments were performed with excitation and emission wavelengths of 320 nm. All experiments were performed at least three times.

### Cell cultures

MCF-7 (wild-type p53) and MDA-MB-231 (mutated p53 – p.R280K) human breast epithelial carcinoma cell lines was obtained from the American Type Culture Collection (ATCC; Manassas, VA, USA). The HCC-70 human breast ductal carcinoma (mutated p53 – p.R248Q) was a donation from National Cancer Institute of Brazil (INCA). Cells were cultured in DMEM containing 4.5 g/L glucose supplemented with 2.0 g/L HEPES, 3.7 g/L sodium bicarbonate, and 10% fetal bovine serum (FBS). Penicillin (100 U/mL) and streptomycin (100 mg/mL) were added to culture plates prior to treatments. Cells were maintained at 37° C in a humidified atmosphere containing 5% CO_2_.

### Immunofluorescence co-localization assays

Cells were grown to 70-80% confluence and treated with resveratrol or its structural analogues pterostilbene and piceatannol for 24 h. Subsequently, the cells were washed twice with PBS (phosphate-buffered saline), fixed with a formaldehyde solution (3.7%) and permeabilized with Triton X-100 (0.5%). The cells were then incubated with 5 mM ammonium chloride for 30 min, and nonspecific antigens were blocked with 3% PBS/BSA (bovine serum albumin) for 2 h. The cells were simultaneously labeled with a mouse monoclonal anti-human p53 protein DO-1 primary antibody (1:200) and an anti-oligomer A11 primary antibody (1:1000) for 2 h at room temperature. Next, the cells were incubated with anti-mouse Texas Red 561 or Alexa Fluor 488 and anti-rabbit IRDye 680 LT-conjugated or Alexa Fluor 546 secondary antibodies, for 1 h at room temperature in a dark chamber. The cells were washed and analyzed using confocal laser scanning microscopy (LSM 510 Meta, Carl Zeiss Inc.).

### Cell proliferation assay

Cell proliferation was assessed using the trypan blue exclusion test of cell viability. MDA-MB-231 cells cultured in a 24-well plate were treated with resveratrol for 24 h. Then, cells were washed with PBS and resuspended with 100 μL of trypsin in 500 μL of 2% DMEM. An aliquot was stained with trypan blue dye (1:1), and the viable cells were immediately counted in a Neubauer’s Chamber using an optical microscope.

### Wound-healing assay

To determine the effect of resveratrol on cell migration, wound-healing assays were performed. MDA-MB-231 cells cultured in a 24-well plate were grown to 50–60% confluence. Cell monolayers were then washed with PBS and scratched with a sterile plastic p10 pipette tip. Wounds were made in triplicate. The scratched cells were removed with two PBS washes, and fresh media containing 2% FBS was added. Cells were treated with resveratrol for 48 h, and images at the zero and final time-points were acquired with a bright-field microscope using a 10x objective. The wound width was measured using ImageJ software.

### Xenograft study

All of the animal experiments were approved by the Ethics Committee of the Institute of Medical Biochemistry of the Health Sciences Center of the Federal University of Rio de Janeiro. Nude athymic mice 4 to 6 weeks of age were purchased from the National Cancer Institute (Rio de Janeiro, RJ). MDA-MB-231 cells were implanted subcutaneously into the right and left flanks (5 × 10^6^ cells/flank) of each animal (*n* = 5). Once palpable tumors (∼220 mm^3^) formed, resveratrol (25 mg/kg body weight) was administered IP daily for 10 consecutive days in the treat animal group. In the control the vehicle was administered (PBS plus 0.2% dimethyl sulfoxide). All of the animals were monitored regularly, and both tumor volume and body weight were recorded weekly. At the end of treatment, the mice were euthanized, and the tumors, lungs and livers were surgically excised. A portion of the tissues was fixed in 10% formalin for histopathological and immunohistochemical analysis.

### Statistical analysis

Results were expressed as the mean ± standard deviation of the mean (S.E.M.). Data were analyzed by the Student’s *t*-test or one-way analysis of variance (ANOVA) using the *post hoc* multiple comparisons Tukey’s test, and *p* ˂ 0.05 was considered statistically significant.

## SUPPLEMENTARY MATERIALS FIGURES


