# Supplementary material for: Resveratrol prevents p53 aggregation in vitro and in breast cancer cells
Source: Oncotarget. 2018 Jun 26;9(49):29112–22. doi: 10.18632/oncotarget.25631 (PMC6044377; doi:10.18632/oncotarget.25631)
Supplement: Supplementary file 1 [file oncotarget-09-29112-s001.pdf]

Resveratrol prevents p53 aggregation *in vitro* and in breast cancer cells

SUPPLEMENTARY MATERIALS

Preparation of BSA aggregates and thioflavin spectroscopy

BSA was dissolved in Tris-HCl 50 mM pH 7.4 buffer to a final concentration of 2.5 mg/ml and filtered through a 0.2-µm membrane. The samples were incubated at 70° C for 90 min without agitation and stored on ice until ThT addition. The samples were mixed with ThT to a final concentration of 20 µM at the moment of measurement. Fluorescence was measured in an ISS-K2 Multifrequency Phase Fluorometer (ISS, Champaign, IL) with excitation and emission wavelengths of 450 and 482 nm, respectively.

Hematoxylin and eosin staining

The formalin-fixed tissues were deparaffinized, gradually dehydrated, embedded in paraffin, subsequently sectioned, and stained with hematoxylin and eosin. The slides were then sealed with Entellan new mounting medium (Merck Millipore), and images were captured using a Nikon Microscope - ECLIPSE at a magnification of 200× and 400×. The liver and lung tissues from both groups were evaluated for the presence of neovascularization and metastasis.

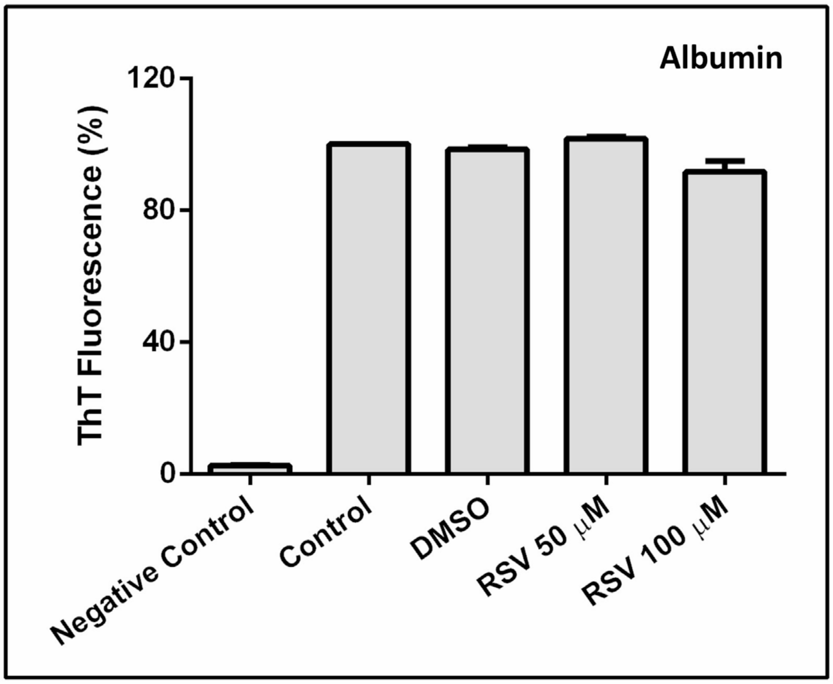

Supplementary Figure 1: Resveratrol does not change BSA thermal aggregation. BSA (2.5 mg/ml) were incubated with resveratrol at 50 and 100 µM at 70° C and the aggregation kinetics were monitored by measuring ThT fluorescence.

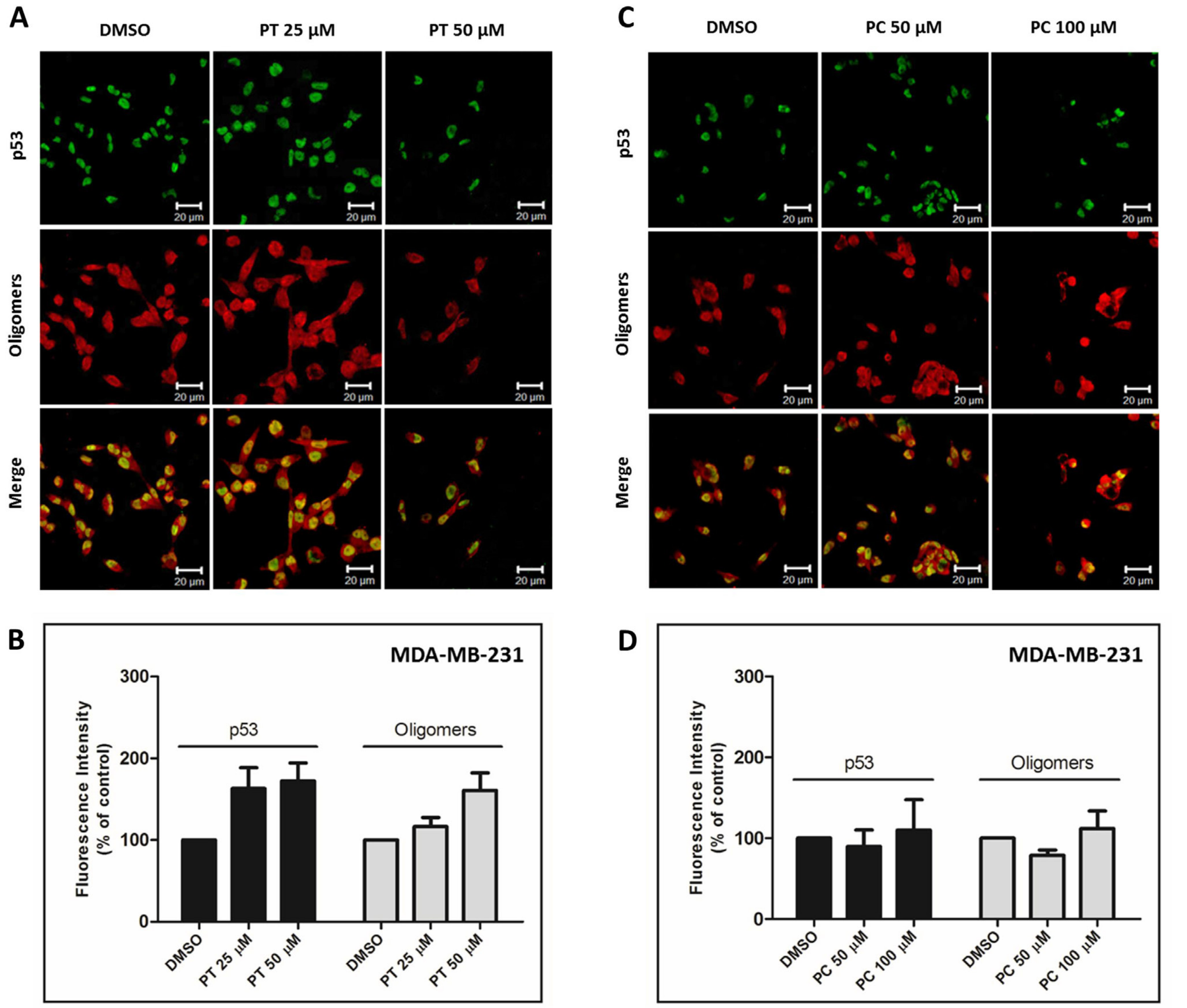

Supplementary Figure 2: Resveratrol derivatives do not prevent p53 aggregation in breast cancer cells. MDA-MB-231 cells were incubated in the presence of pterostilbene 25 and 50 μM (**A**) and piceatannol 50 and 100 μM (**B**) for 24 h. Cells were simultaneously labeled with a mouse monoclonal anti-human p53 protein DO-1 primary antibody (1:200) and an anti-oligomer A11 (1:1000) primary antibody, as indicated. Next, cells were incubated with Texas Red 561- and IRDye 680LT-conjugated secondary antibodies at room temperature in a dark chamber. Finally, cells were washed and analyzed using confocal laser scanning microscopy. Total fluorescence intensity quantification of panels A and B was performed using ImageJ software, version 1.43r (NIH, USA) (**C** and **D**, respectively).

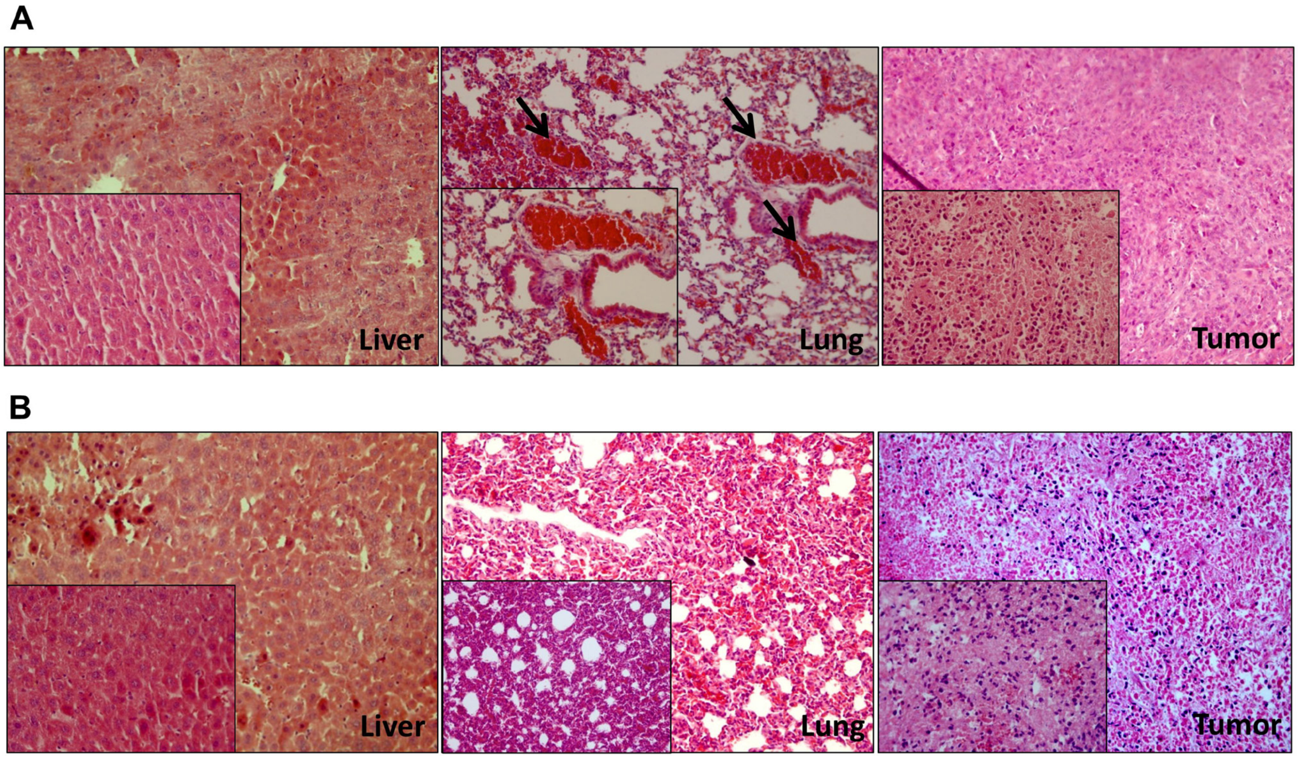

Supplementary Figure 3: Effect of resveratrol in the liver, lung and tumor tissues of a nude mouse xenograft model of breast cancer using MDA-MB-231 cells. Nude mice with identified tumors were treated with vehicle or resveratrol (25 mg/kg daily IP for 10 days). (**A**) Representative images demonstrating the excised tissues from both groups. (**B**) The tissues and tumors of animals were evaluated by staining with hematoxylin and eosin. More blood vessels were observed in the lungs of the untreated group, possibly for the establishment of metastasis. Arrows indicate angiogenesis, or increased blood vessel number and size. Images: 200×. Inset: 400×.
